# Molecular Link between Glo-1 Expression and Markers of Hyperglycemia and Oxidative Stress in Vascular Complications of Type 2 Diabetes Mellitus

**DOI:** 10.3390/antiox12091663

**Published:** 2023-08-23

**Authors:** Nida Ali Syed, Attya Bhatti, Peter John

**Affiliations:** 1 Department of Healthcare Biotechnology, Atta-ur-Rahman School of Applied Biosciences, National University of Sciences and Technology, Islamabad 44000, Pakistan; nsyed.phdabs11asab@student.nust.edu.pk (N.A.S.); pjohn@asab.nust.edu.pk (P.J.); 2 Department of Internal Medicine, Faculty of Health, Medicine and Life Science, Maastricht University, P.O. Box 616, 6200 MD Maastricht, The Netherlands

**Keywords:** Glyoxalase-1, VCAM, TXNIP, Type 2 Diabetes Mellitus, vascular complications, hyperglycemia, inflammation, oxidative stress

## Abstract

Chronic hyperglycemia and oxidative stress in Type 2 Diabetes Mellitus trigger cellular dysfunction via the formation of Advanced Glycation End Products (AGEs), resulting in dicarbonyl stress. Glyoxalase-1 (Glo-1) is the main defense against dicarbonyl stress. The aim of this study was to explore any cross-talk between Glo-1 and markers of hyperglycemia and oxidative stress. The siRNA-mediated downregulation of Glo-1 was performed in human microvascular endothelial cell line (HMEC-1). A Glo-1 transgenic rat model was developed. Glo-1 activity, as determined spectrophotometrically, and methylglyoxal were quantified using UPLC-MS/MS and the expression of representative markers of hyperglycemia and oxidative stress was performed using quantitative real-time PCR. A significant increase in the expression of Vascular Cell Adhesion Molecule-1 (VCAM-1) was observed in the case of the siRNA-mediated downregulation of Glo-1 in the microvasculature model under hyperglycemic conditions (*p*-value < 0.001), as well the as overexpression of Glo-1 in the macrovasculature (*p*-value = 0.0125). The expression of thioredoxin interacting protein (TXNIP) was found to be significantly upregulated in wildtype diabetic conditions vs. Glo-1 transgenic control conditions (*p*-value = 0.008), whereas the downregulation of Glo-1 had no impact on TXNIP expression. These findings substantiate the role of VCAM as an important marker of dicarbonyl stress (represented by Glo-1 downregulation), as well as of hyperglycemia, in diabetic vascular complications. Our findings also suggest a potential feedback loop that may exist between Glo-1 and TXNIP, as the highest expression of TXNIP is observed in cases of wildtype diabetic conditions, and the lowest expression of TXNIP is observed when Glo-1 transgene is being expressed in absence of dicarbonyl stress.

## 1. Introduction

Type 2 Diabetes Mellitus (T2DM) is a heterogeneous metabolic disorder primarily characterized by chronic hyperglycemia and insulin insufficiency. It is fast attaining the status of an epidemic and it is estimated that it will affect 1 in every 10 individuals by 2040 globally. The rapid rise in the prevalence of T2DM has become a significant global health issue, largely because of its associated complications, treatment expenses, and shortened life expectancy [1]. The vascular complications of diabetes, just like the disease itself, are complex multifactorial conditions with both genetic and environmental influences. These complications stem from diabetes-associated long-term damage to both large and small blood vessels throughout the body, referred to as macrovascular and microvascular damage, respectively. The most common microvascular complications of T2DM include retinopathy, nephropathy, neuropathy, and cataract, while macrovascular complications include atherosclerosis, coronary heart disease, and peripheral vascular and cerebrovascular diseases in diabetic patients [2].

Key pathological hallmarks of T2DM range from chronic hyperglycemia to oxidative stress, and insulin insufficiency. In addition, chronic tissue inflammation has also been implicated in the pathogenesis of diabetes and its associated micro- and macrovascular complications. Downstream consequences of elevated glucose levels transcend across multiple pathophysiological processes, such as nonenzymatic protein glycation, oxidative stress, and inflammation, which lead to biochemical dysfunction associated with the chronic development of microvascular damage [3]. Chronic hyperglycemia is known to induce vascular inflammation and oxidative stress in the endothelium, leading to endothelial barrier dysfunction and culminating in diabetes-associated vasculopathies [4]. Insulin resistance and endothelial dysfunction have been observed to precede the event of overt hyperglycemia.

Endothelial dysfunction is represented by altered cellular signaling, increased oxidative stress, proinflammatory activation and mitochondrial dysfunction. It is driven by hyperglycemia as mentioned above [4]. Hallmarks of endothelial cell dysfunction include the accumulation of reactive dicarbonyl metabolites, also referred to as dicarbonyl stress, the upregulation of inflammatory signaling and secretion of inflammatory cytokines, and the expression of adhesion molecules and apoptosis [5]. 

Chronic hyperglycemia is a breeding ground for the spontaneous, nonenzymatic formation of Advanced Glycation End Products, herein abbreviated to AGEs, via the infamous Maillard reaction. The Maillard reaction is defined as the formation of adducts between reactive carbonyls in glucose, fructose, and their metabolites, such as methylglyoxal or deoxyglucosone, with amino groups in protein, DNA, and lipids. This reaction has been implicated as a root cause of several evils in the form of diabetes-associated complications and comorbidities [6]. Methylglyoxal (MGO) is a highly reactive dicarbonyl oxoaldehyde that forms during glycolysis and acts as a precursor for AGEs. MGO is detoxified by Glyoxalase-1 (Glo-1), the primary cellular defense against dicarbonyl stress responsible for neutralizing >99% of MGO, thereby reducing MGO overload and limiting AGE formation [7]. Previous studies have showed that altered Glyoxalase-1 activity in the blood is related to the onset of diabetic complications [3]. Another study conducted in diabetic mice showed that an enhanced glyoxalase system with a concomitant reduction in methylglyoxal-dependent protein glycation can prevent the onset of vascular complication in diabetes [8]. Dicarbonyl stress is defined as a state of dysfunction marked by an accumulation of methylglyoxal (MGO) and other reactive oxoaldehydes as a consequence of their increased formation or reduced detoxification by Glyoxalase-1 [9,10].

Glo-1 is transcriptionally regulated by a redox-sensitive transcription factor Nrf2 (Nuclear Factor (Erythroid-derived 2)-like 2). This regulates the transcription of various detoxification and antioxidant enzyme genes (e.g., Glo-1, SOD-2) and plays an important role in protection against environmental stresses, reactive oxygen species and reactive nitrogen species [11]. The depletion of Nrf2 in a streptozotocin (STZ)-induced mouse diabetes model resulted in increased renal oxidative and nitrosative stress. Results from another study have showed that the administration of Nrf2 inducers in an STZ-induced diabetes model has shown to be beneficial for the prevention and treatment of diabetic complications [12]. 

The overexpression of SOD-2, also known as manganese superoxide dismutase (MnSOD), under the transcriptional regulation of Nrf-2; like Glo-1, represents the primary line of defense against oxidative stress caused by reactive oxygen species (ROS), generated on the matrix side of the inner mitochondrial membrane due to hyperglycemia [13,14]. 

Sirtuin-1 (SIRT1) is a key member of the family of NAD^+^-dependent class III histone deacetylases. It is an important regulator of metabolic adaptation, DNA repair, cell survival, and the oxidative stress response. Recent studies have shown that SIRT1 is a prerequisite for the regulation of cellular glycative stress via the regulation of the glyoxalase system [15].

The combined effect of hyperglycemia and oxidative stress is responsible for the activation of the NLRP3 (NACHT (NOD-like receptor), LRR, and PYD domain-containing protein-3) inflammasome. Inflammasomes are cytosolic macromolecular complexes capable of detecting and eliciting an inflammatory response to a range of pathogenic signals; this includes aberrant glucose metabolism and oxidative stress [16]. The mitochondrial ROS-TXNIP-NLRP-3 biological axis has recently come to light due to its role in the pathogenesis of diabetic complications. The NLRP3 inflammasome comprises a sensing protein, NLRP3, an adaptor protein, the apoptosis-associated speck-like protein containing a caspase recruitment domain (ASC), and an effector protein, pro-caspase-1. [17]. 

TXNIP (thioredoxin interacting protein) is an endogenous antioxidant like SOD-2, which is overexpressed under hyperglycemic and diabetic conditions [18]. The excessive production of ROS triggers the dissociation of TXNIP from thioredoxin (TRX), following which it associates with the NLRP-3 inflammasome via a leucine-rich repeat sequence to form an active inflammasome complex [17]. Following its assembly, the NLRP3 inflammasome coordinates the inflammatory response via caspase-1 activation and IL-1β maturation [19].

Caspase-1 is the most common and most well-studied inflammation-mediated caspase. It initiates the inflammatory response through the maturation of pro-inflammatory cytokines, such as interleukin-1β (IL-1β) and IL-18. Caspase-1 also regulates the expression of key hyperglycemia-induced genes, such as IL-1β, SIRT-1, and NF-Kβ, that have been implicated in diabetes-associated complications [20].

Interleukin 1β (IL-1β) is a prominent proinflammatory cytokine that is critical for the initiation and sustenance of inflammation-induced organ dysfunction in T2DM [21]. The expression of IL-1β is known to be upregulated in T2DM as a marker of hyperglycemia-induced inflammation, which leads to substantial damage to endothelial cells, thereby contributing to vascular complications in T2DM [22]. One of the mechanisms by which IL-1β activates the endothelium is via the upregulation of the adhesion molecules VCAM (Vascular Cell Adhesion Molecule) and ICAM (Intercellular Adhesion Molecule), which are regarded as markers of inflammation and are associated with diabetic complications [23]. 

Mechanisms underlying hyperglycemia-induced cellular dysfunctions, such as endothelial dysfunction, remain incompletely understood at the molecular level. Despite being acknowledged as the foremost cellular defense against dicarbonyl stress, Glo-1 remains an elusive entity in the bigger picture, representing one of the major players of T2DM pathogenesis in various biochemical pathways. The aim of this study was to develop an understanding of how Glo-1 interacts with other genes associated with inflammation, oxidative stress, and the micro- and macrovascular complications of Type 2 Diabetes Mellitus. More specifically, we wanted to study whether the down- and upregulation of Glo-1 expression exerts any impact on the expression of putative genes implicated in inflammation, oxidative stress, and endothelial dysfunction, under normoglycemic and hyperglycemic conditions in the micro- and macrovessels, respectively. 

## 2. Materials and Methods

### 2.1. In Vitro Model of Dicarbonyl Stress

An in vitro model of dicarbonyl stress was developed using the siRNA-mediated knockdown of the Glo-1 gene in the HMEC-1 cell line. 

#### 2.1.1. Cell Culture

HMEC-1 (American Type Culture Collection Cat# CRL-3243, Manassas, VA, USA; RRID: CVCL_0307) (provided by Prof Casper G. Schalkwijk) is an adherent cell line derived from dermal microvascular endothelial cells. It was grown in T-75 culture flasks (Corning Incorporated, Corning, NY, USA) that had been coated with 1% gelatin solution prior to cell seeding, using MCDB 131 complete culture medium supplemented with 100 units/mL Penicillin, 100 µg/mL Streptomycin and 2 mM L-Glutamine, as well as 10% inactivated Fetal Calf Serum, 0.1% hydrocortisone and 1% Endothelial Cell Growth Factor and maintained at a temperature of 37 °C with 5% atmospheric CO_2_. 

#### 2.1.2. siRNA-Mediated Knockdown of Glo-1 Expression

For all siRNA transfection experiments, HMEC-1 cells were seeded at a density of 1.0 × 10^5^ cells/mL in a 6-well plate for 24 h. The siRNA-mediated inhibition of Glo-1 was performed using Glo-1 Silencer^®^ Predesigned siRNA (Cat # AM16708; Ambion, Life Technologies, Carlsbad, CA, USA) with the sense siRNA having the sequence GCUACACUUGAGCUGACACtt and the antisense siRNA having the sequence GUGUCAGCUCAAGUGUAGCtt (5′ to 3′). A universal scrambled siRNA Silencer^®^ Select Negative Control #1 siRNA (Cat # 4390843) (ThermoFisher Scientific, Waltham, MA, USA) was used as the negative control and Lipofectamine™ 2000 (Invitrogen, Waltham, MA, USA) was used as the transfection reagent. Both siRNA and Lipofectamine were diluted in Opti-MEM™ reduced serum medium (Gibco, ThermoFisher Scientific, Waltham, MA, USA) and transfection was performed according to manufacturer’s instructions.

During transfection, cells were supplemented with ‘transfection medium’ that was composed of MCDB-131 medium, inactivated Fetal Calf Serum, ECGH, Hydrocortisone and Glutamine. The difference between the culture medium and the transfection medium was the presence of antibiotics, i.e., Penicillin and Streptomycin, which are not added as they have been reported to interfere with siRNA transfection, according to the manufacturer’s instructions. 

Following siRNA/scrambled siRNA treatment, cells were treated with normal and high glucose concentrations for 24 h (5 mM and 25 mM, respectively, prepared in complete culture medium). The spectrophotometric determination of Glo-1 activity, the quantification of MGO and its adducts, and gene-expression assays were performed 48 h after siRNA transfection and 24 h after the normal- or high-glucose treatment.

#### 2.1.3. Spectrophotometric Determination of Glo-1 Activity

Glo-1 activity assay is a biochemical assay for the spectrophotometric determination of Glyoxalase-1 enzymatic activity at 240 nm, expressed as the amount of S-D-Lactoylglutathione in nmol/min/mg of protein formed. It was performed according to the protocol by McLellan and Thornalley [24]. Briefly, the following reaction takes place:

$$\mathrm{Methylglyoxal}\;+\;\mathrm{Glutathione}\left(\mathrm{reduced}\right)↔\mathrm{Hemithioacetal}\;\overset{\mathrm{Glyoxalase}\text{-}1}{\to }s\text{-}D\text{-}\mathrm{Lactoylglutathione}$$

The concentration of s-D-Lactoylglutathione formed in a given reaction well is measured at 240 nm, and this value is used to determine the activity of Glyoxalase-1 present in the sample being tested, which is expressed as the amount of s-D-Lactoylglutathione in nmol/min/mg protein formed ɛ.

This calculation was based on Lambert Behr’s formula:

E = ɛ × [s-D-Lactoylglutathione] × I

where ɛ is the absorbance constant of s-D-Lactoylglutathione (3.37 mM/cm), and I is the path length (0.588 cm for a well of a 96-well plate).

The concentration of protein in cell lysate samples was determined using the Pierce™ Bicinchoninic acid (BCA) Protein Assay Kit (ThermoFisher Scientific™) following the manufacturer’s protocol.

### 2.2. In Vivo Model of Dicarbonyl Stress

The transgenic rat model expressing Glyoxalase-1 was developed using the human Glo-1 transgene construct. The entire coding sequence of the Glo-1 cDNA was cloned into the EcoR1 site of pBsCAG-2. The Glo-1 transgene was isolated by the digestion of pBsCAG-2-containing Glo-1 cDNA with KpnI and SacI, respectively, and microinjected directly into the pronuclei of fertilized Wistar rat eggs, followed by transfer into the oviducts of pseudopregnant rats. 

Diabetes was induced in wildtype (n = 9) and transgenic Glo-I rats (n = 8) by administering an intravenous injection of streptozotocin (STZ, 65 mg/kg body weight). Weight- and age-matched control rats (wildtype, n = 9), and transgenic Glo-I rats (n = 8) were not induced with diabetes. 

All animal studies were carried out in accordance with the Guide for the Care and Use of Laboratory Animals of the National Institutes of Health and the ARRIVE guidelines and reviewed and approved by the ethics committee for animal care of Maastricht University, as well as the National University of Sciences and Technology (ethical approval reference number ASAB-IRB-115; dated 19 December 2017). 

### 2.3. Gene Expression

Gene-expression analysis was conducted using Real-time quantitative PCR. RNA was extracted using the TRIzol reagent (Invitrogen) and RNA concentration and purity were determined using the Nanodrop Spectrophotometer. cDNA synthesis was performed using the iScript™ cDNA Synthesis kit (Bio Rad, Hercules, CA, USA), containing Reverse Transcriptase enzyme, RNA inhibitors, oligo dT primers and RNA template, using the thermocycle profile 25 °C for 5 min, 46 °C for 20 min, 95 °C for 1 min and 4 °C for 0.5 min. qPCR was performed using the SensiMix™ SYBR^®^ master mix (Meridian Bioscience, Cincinnati, OH, USA), 6 µM each of forward and reverse primer, and 10 ng cDNA template. 

The following gene panel was used for gene expression analysis in both models of dicarbonyl stress: Glo-1, Nrf-2, SIRT-1, SOD-2, IL-1β, TXNIP, Caspase-1, NLRP-3, I-CAM, and V-CAM. β_2_-Microglobulin and Cyclophilin were used as housekeeping genes for the normalization of gene expression. Primer sequences used for gene-expression profiling are given in Table 1. The following protocol was used for real-time PCR: an initial activation step for 10 min at 95 °C, and amplification step of 40 cycles (10 s at 95 °C and 40 s at 60 °C) followed by 95 °C for 1 min, 60 °C for 1 min, and a melt curve and plate read step with 0.5 °C increments from 60 °C to 95 °C for 10 sec. The fold change in mRNA expression was calculated using the comparative 2^−ΔΔCt^ method [20].

### 2.4. Data Analysis

All data were analyzed for statistical significance using GraphPad Prism software version 8.0.2.263. Data are expressed as mean ± S.E.M or mean ± S.D. A *p*-value less than 0.05 was considered significant.

## 3. Results

### 3.1. Effect of siRNA-Mediated Downregulation of Glo-1 on Markers of Hyperglycemia and Oxidative Stress

#### 3.1.1. Spectrophotometric Determination of Glyoxalase 1 Activity

**Figure 1:**
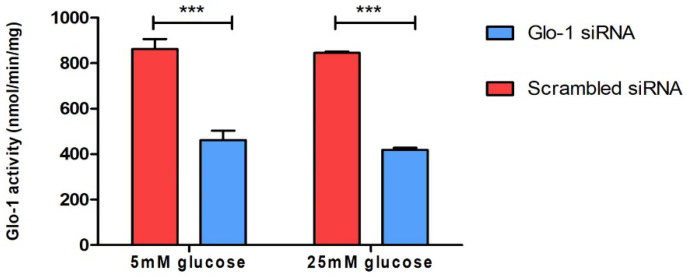
Spectrophotometric determination of Glyoxalase-1 activity. Data are represented as mean ± SD (n = 3); significance *** *p* < 0.0001. Statistical significance was determined using two-way ANOVA with Bonferroni post hoc test.

To understand the effect of the downregulation of Glyoxalase-1 expression on markers of various aspects of endothelial dysfunction, HMEC-1 cells were cultured and transfected as described in the methodology. The siRNA-mediated knockdown of Glo-1 was followed by 24 h of treatment in normoglycemic (5 mM) and hyperglycemic (25 mM) conditions in vitro. The spectrophotometric determination of Glyoxalase-1 activity at 240 nm was performed after 48 h of siRNA transfection. It showed a significant downregulation of Glyoxalase-1 activity (approximately 50%) following siRNA inhibition (Figure 1). Glo-1 activity was measured in three experimental replicates and is expressed as mean ± S.D.

#### 3.1.2. Quantification of MGO and Its Derived Adducts

**Figure 2:**
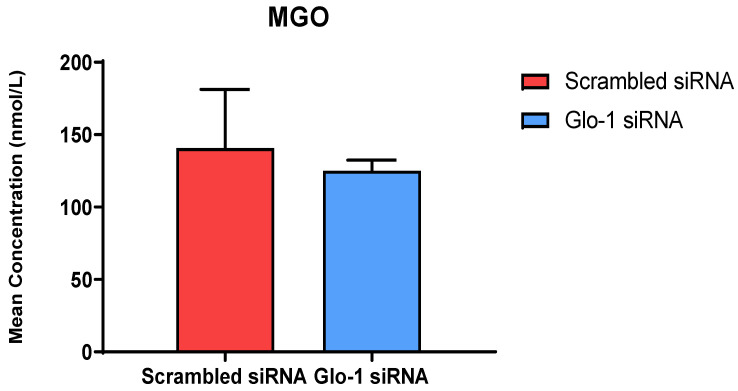
Quantification of MGO was performed after 48 h of transfection with Glo-1 siRNA and Scrambled siRNA via UPLC-MS/MS of cell culture supernatant samples. Data is statistically analyzed by Two-way ANOVA and represented as Mean ± SD (n = 3).

The quantification of MGO in cell culture supernatant was performed after 48 h of transfection with Glo-1 siRNA and Scrambled siRNA followed by 24 h treatment with 5 mM and 25 mM glucose, respectively using UPLC-MS/MS. No significant difference was observed in the mean concentration of MGO between the Glo-1 siRNA and control siRNA transfected groups (Figure 2).

#### 3.1.3. Quantitative Gene Expression of Markers of Hyperglycemia and Oxidative Stress

**Figure 3:**
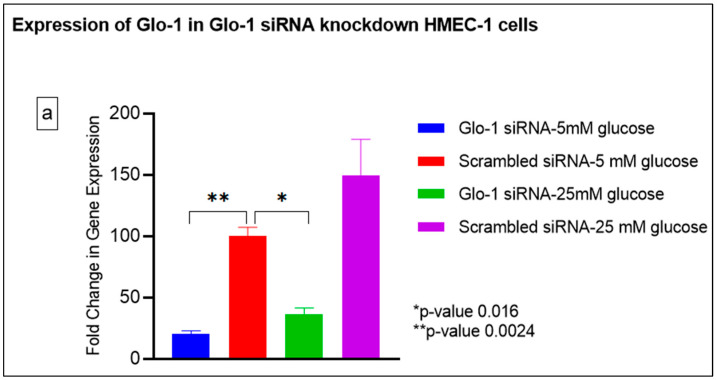

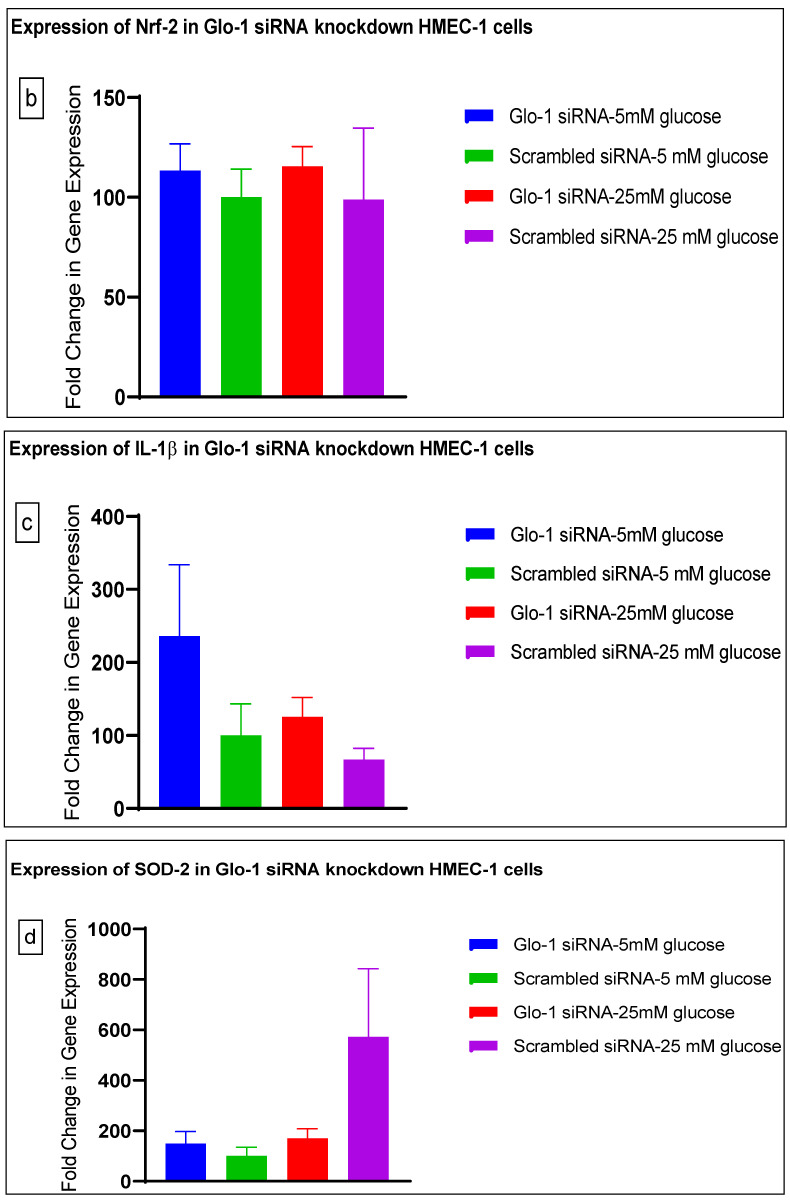

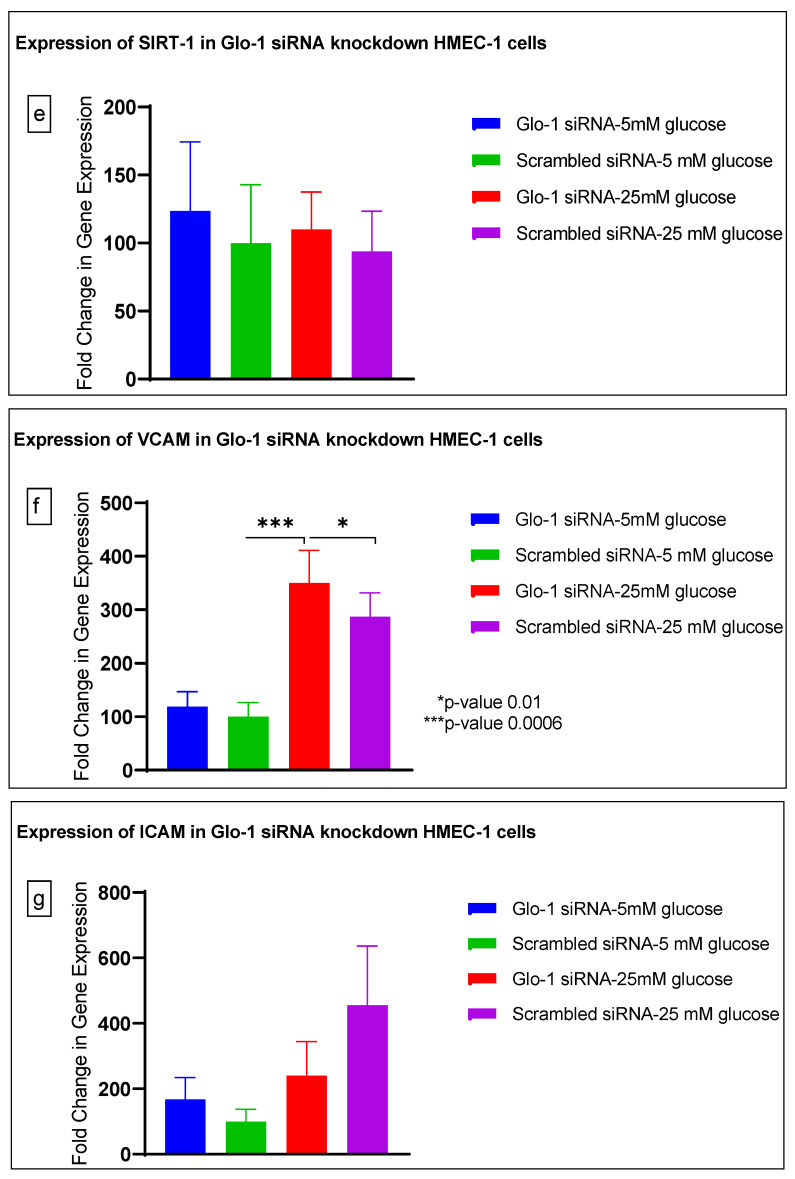

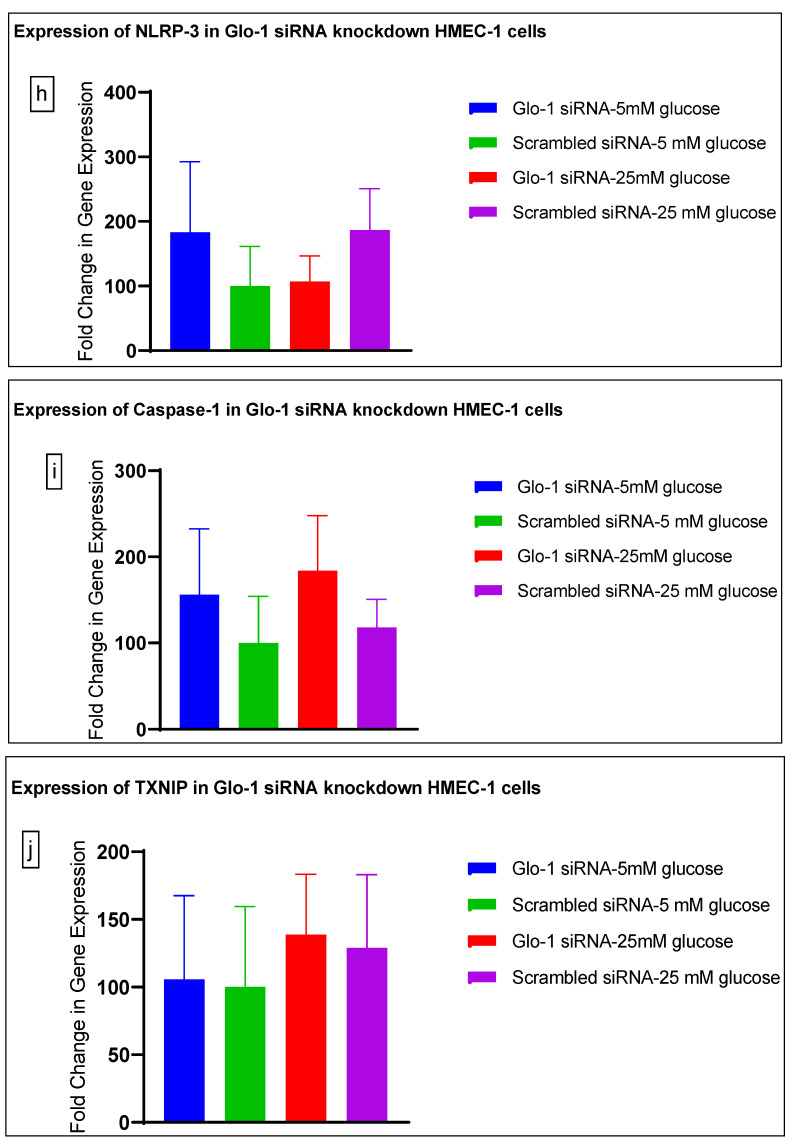
(**a**–**j**) Fold change in mRNA expression of Glo-1, Nrf-2, IL-1β, SOD-2, SIRT-1, VCAM, ICAM, Caspase-1, TXNIP and NLRP-3. Data were statistically analyzed using one-way ANOVA followed by Dunnett’s post hoc test and represented as mean ± S.E.M.

Gene expression of Glyoxalase-1 gene was determined using real time quantitative PCR as a means to confirm successful siRNA mediated downregulation of Glo-1 versus control/scrambled siRNA (Figure 3a–j); which was significantly reduced in the Glo-1-siRNA-transfected cells compared to the control siRNA (*p*-value < 0.001) (Figure 3a). In addition, a significant upregulation in the expression of VCAM (*p*-value < 0.001) was seen in the experimental group experiencing Glo-1 siRNA knockdown followed by the high-glucose treatment, compared to the control/scrambled siRNA-treated cells (Figure 3f). Similarly, a significant difference was also observed in the expression of VCAM between the Glo-1 siRNA followed by 25 mM glucose vs. the scrambled (Control) siRNA followed by 25 mM glucose treatment groups (*p*-value = 0.01). All qPCR reactions were performed after 48 h of transfection with Glo-1 siRNA. The experiment was performed at least thrice, in triplicate; data are represented as the mean ± SEM (N = 4; n = 9) and were statistically analyzed using one-way ANOVA followed by Dunnett’s post hoc test for multiple comparisons between experimental groups; a *p*-value < 0.05 was considered significant.

### 3.2. Effect of Upregulation of Glo-1 Expression on Markers of Hyperglycemia and Oxidative Stress

**Figure 4:**
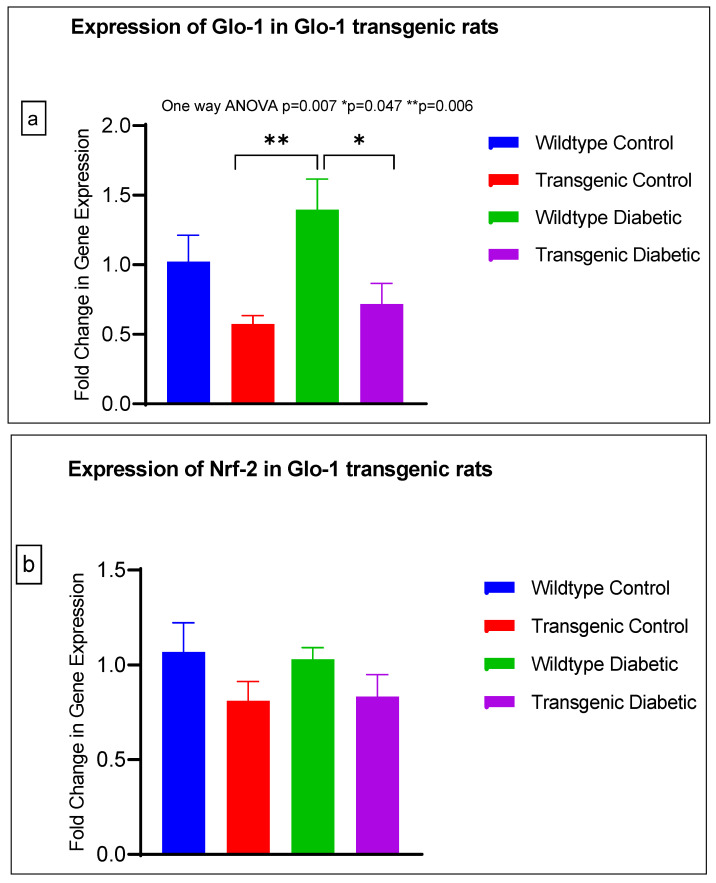

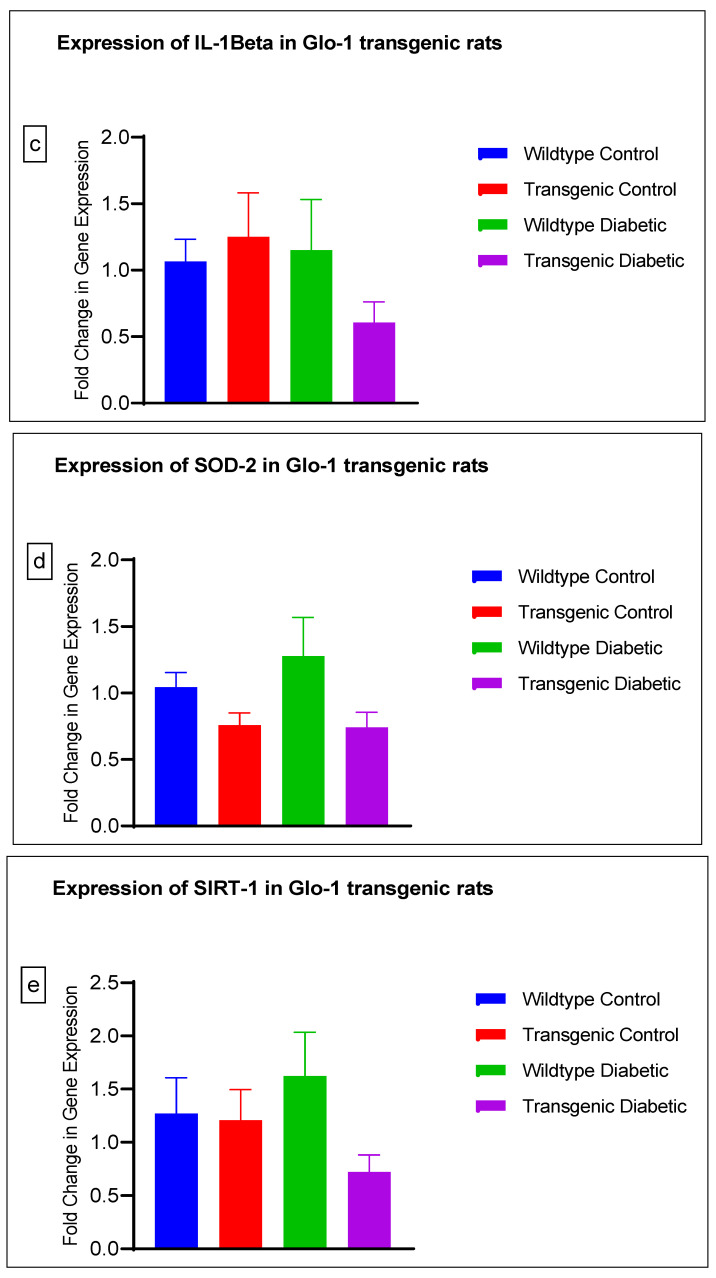

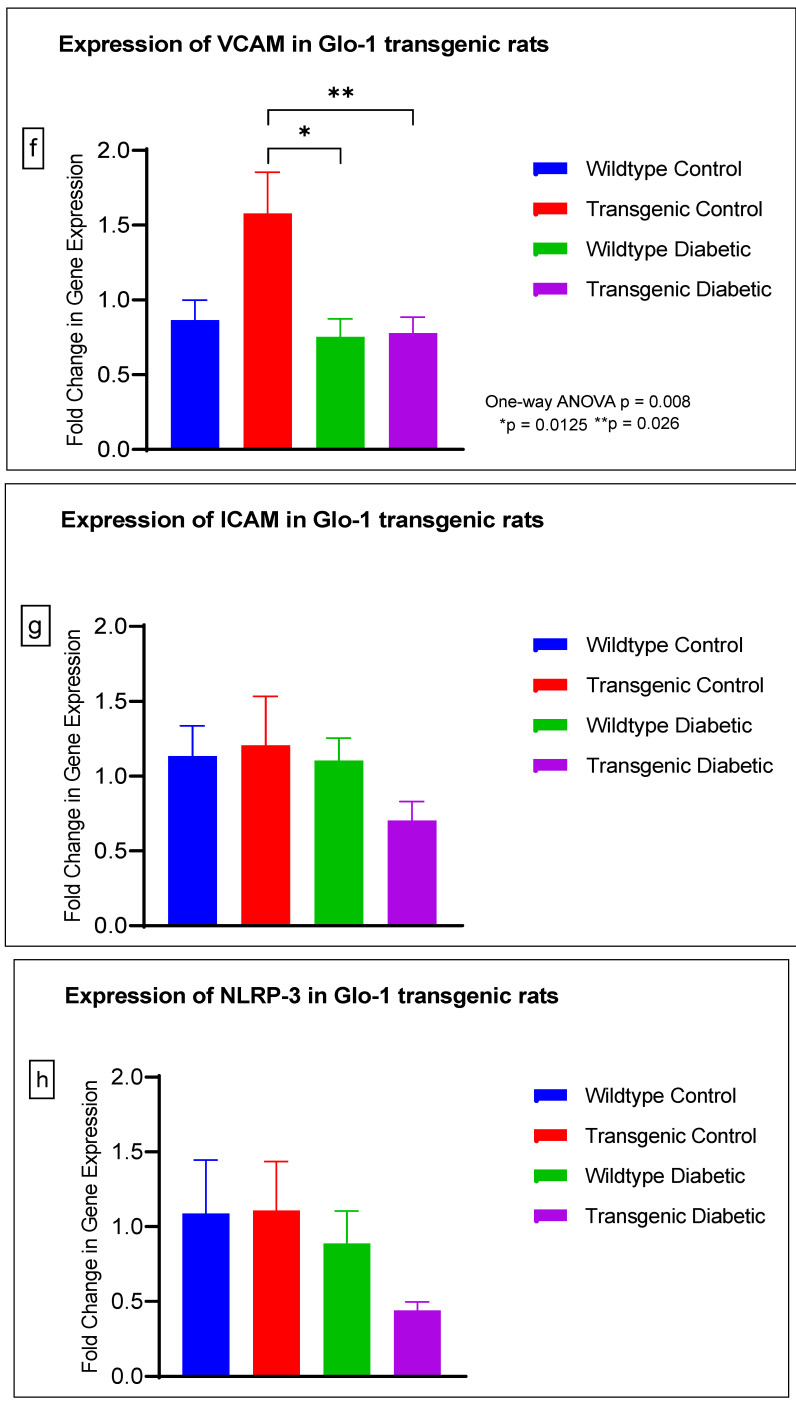

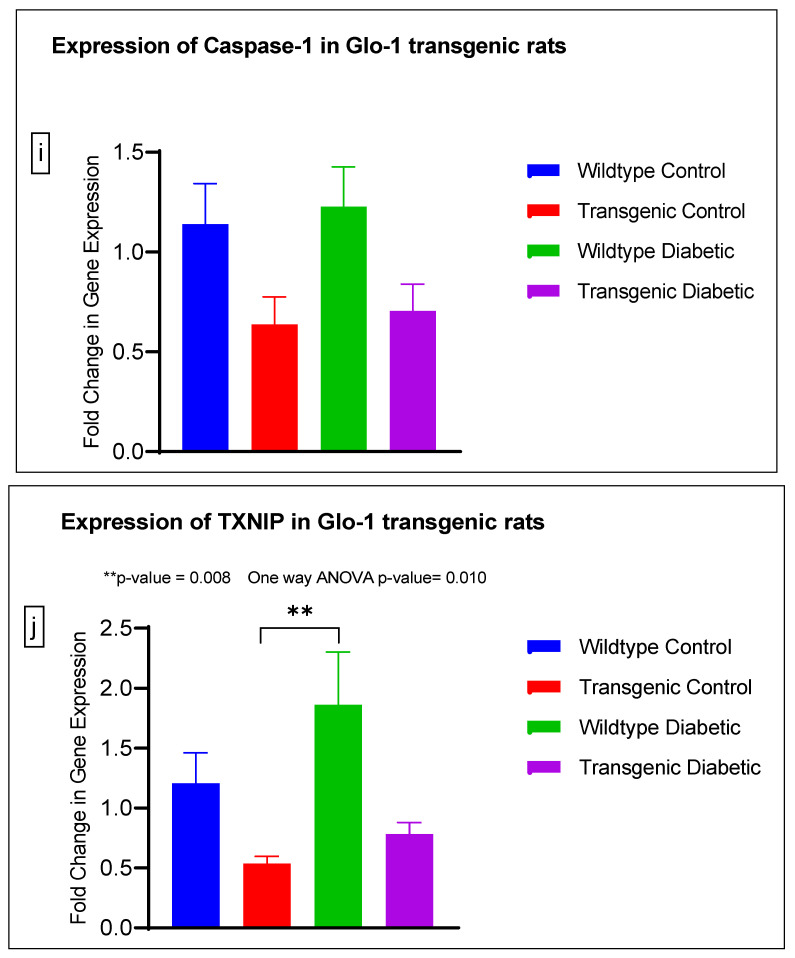
(**a**–**j**) Fold change in mRNA expression of Glo-1, Nrf-2, IL-1β, SOD-2, SIRT-1, VCAM, ICAM, Caspase-1, TXNIP and NLRP-3. Data were statistically analyzed using one-way ANOVA followed by Tukey’s post hoc -test and represented as mean ± S.E.M.

The gene expression of various markers of endothelial dysfunction was determined using real-time quantitative PCR in transgenic Glo-1 rats divided into four experimental groups: wildtype control/healthy rats, wildtype diabetic rats, transgenic control rats and transgenic diabetic rats (n = 7 ± 2) (Figure 4a–j). A one-way ANOVA with Tukey’s post hoc analysis for multiple comparison analysis across various experimental groups was used to determine the statistical difference between the target gene expressions of the experimental groups.

A significant difference was observed between the expressions of the Glo-1 wildtype diabetic and transgenic control groups (*p*-value = 0.006) and between those of the wildtype diabetic and transgenic diabetic rats (*p*-value = 0.047) (Figure 4a). 

There was no significant difference in the expression of Nrf-2, IL-1β, SOD-2, SIRT-1, ICAM, Caspase-1 and NLRP-3. A significant difference in the expression of VCAM was observed between transgenic control and wildtype diabetic rats (*p*-value = 0.0125), as well between the transgenic control and transgenic diabetic groups (*p*-value = 0.026) (Figure 4f). In addition, a significant difference was observed in the expression of TXNIP between the transgenic control and wildtype diabetic groups (*p*-value = 0.008) (Figure 4j). 

## 4. Discussion

Hyperglycemia is the primary defining characteristic of Type 2 Diabetes Mellitus and a major risk factor for endothelial dysfunction and the subsequent development of vascular complications. It induces β-cell and vascular damage due to abnormal glucose uptake in skeletal muscles, the aberrant hormonal regulation of glucose metabolism, and weakened β-cell secretory function. In 2001, Michael Brownlee presented an elegant unifying theory recognizing four key biochemical pathways that mediate hyperglycemia-induced micro- and macrovascular complications in T2DM: increased polyol pathway flux, the increased formation of Advanced Glycation End products (AGEs), increased hexosamine pathway activation and the activation of Protein Kinase C (PKC) isoforms. One hyperglycemia-driven process underlies all four of these pathological pathways: the overproduction of superoxide ions by the mitochondrial electron transport chain [14].

Elevated glucose levels in endothelial cells cause oxidative stress due to the increased production of mitochondrial ROS and the nonenzymatic glycation of proteins, and glucose autoxidation and insulin insufficiency propelling the accumulation of AGEs have been observed in endothelial dysfunction preceding the pathogenesis of diabetes-associated micro- and macrovascular complications [25]. Furthermore, alterations in the Renin–Angiotensin system, oxidative stress and reduced Nitric oxide bioavailability induce vascular inflammation in the endothelium, leading to endothelial dysfunction, vessel wall remodeling and altered vasoreactivity, culminating in diabetes-associated vasculopathies [26]. 

In our study, we explored both aspects of Glo-1 expression, upregulation and downregulation, in an experimental representation of both the micro- and macrovasculature, under normal—(5 mM) and high-glucose (25 mM) conditions. The HMEC-1 cell line was representative of the endothelial cells in the microvasculature, whereas mesenteric arteries from the Glo-1-overexpressing transgenic rat model represented the macrovasculature. The siRNA-mediated inhibition of Glo-1 was first confirmed using a spectrophotometric determination of Glyoxalase-1 activity, whereby an inhibition of approximately 50% was observed (*p*-value < 0.0001). However, post-treatment with normal (5 mM) or high (25 mM) glucose concentrations had no impact on the siRNA-mediated inhibition of Glo-1 activity. 

The levels of MGO were quantified using a highly sensitive technique UPLC-MS/MS in cell culture supernatant samples, and no change was observed under the timepoint and glucose concentration that we used in our experiments. MGO is a highly unstable, endogenous by-product of the normal metabolism of carbohydrates, lipids, and proteins. It readily reacts in vivo, with basic phospholipids and nucleotides, especially lysine and arginine residues of proteins, leading to the formation of advanced glycation end products (AGE). It has been implicated in the pathogenesis of various metabolic, inflammatory, and aging-related diseases. Its accumulation results in dicarbonyl stress, causing an irreversible loss of protein function, and contributing to oxidative stress [27].

Chemically, MGO is an abundant, electrophilic byproduct of metabolic flux produced intracellularly at a concentration of 1–4 µM [28]. However, due to its small size, it rapidly permeates through cell membranes into the extracellular space and vice versa [29]. With a short biological half-life, it is likely that a higher actual amount of MGO is produced than that being experimentally estimated [30]. Due to the highly reactive nature of MGO, its direct quantification is difficult; instead, the quantification of MGO-derived AGEs can provide an insight into MGO production [31]. 

The Glyoxalase system represents the primary line of enzymatic defense against dicarbonyl stress by detoxifying MGO; with secondary defense provided by Aldoketo reductases (AKRs) and Aldehyde dehydrogenases (ADHs) in absence of Glo-1 [32]. It is comprised of Glyoxalase-1 (Glo-1), Glyoxalase-2, and a catalytic amount of reduced glutathione. It is the foremost endogenous defense system that detoxifies >99% of MGO thereby reducing MGO overload and restricting AGEs formation. MGO accumulation and AGEs formation in the state of dicarbonyl distress is a direct consequence of chronic hyperglycemia which perpetuates physiological insufficiency of the Glyoxalase system via reduced Glo-1 transcription and activity. Reduced capacity of Glo-1 to detoxify MGO has been observed in the case of endothelial dysfunction that precedes the pathogenesis of diabetes-associated micro- and macrovascular complications [2,33]. 

Overexpression of Glo-1 is said to have a protective effect against reactive oxygen species (ROS), glucose-driven apoptosis, and dysfunction arising from angiogenesis and diabetes [27,28]. At present, it is not known how and if Glo-1 interacts with other putative hyperglycemia-induced genes and how this interaction may play out in the onset and progression of micro- and macrovascular complications of T2DM. 

Previous studies have shown that altered Glyoxalase-1 activity in the blood is related to the onset of diabetic complications [3]. Another study conducted in diabetic mice showed that an enhanced glyoxalase system with a concomitant reduction in methylglyoxal-dependent protein glycation can prevent the onset of vascular complication in diabetes [8].

Here, we have shown that the siRNA-mediated downregulation of Glo-1 leads to an upregulation in the expression of VCAM under hyperglycemic conditions (*p*-value = 0.003). However, no change was observed in the expression of SIRT-1, ICAM, antioxidant gene SOD-2 and its transcription factor Nrf-2, or the NLRP-3 inflammasome and its associated proteins Caspase-1 and TXNIP. 

In order to understand the impact of Glo-1 upregulation as a comparative analysis to Glo-1 downregulation, a Glo-1-overexpressing transgenic rat experimental model was developed. In this model, we observed that the expression of TXNIP was upregulated in wildtype diabetic rats (*p*-value = 0.01) in comparison to the transgenic control group. In the case of VCAM, a significant difference in expression was observed in the transgenic control vs. the wildtype diabetic group (*p*-value = 0.0125) and the transgenic control vs. the transgenic diabetic group (*p*-value = 0.026), respectively. 

VCAM-1 is a cellular adhesion molecule expressed on the surface of ‘activated’ endothelial cells in response to hyperglycemia-induced oxidative stress and cytokine production in T2DM. It is a member of the immunoglobulin superfamily that interacts with its ligand, integrin VLA-4 (Very Late Antigen-4) (CD49d/CD29), which is expressed on the surface of numerous leukocytes [34]. VCAM acts as a mediator between chronic inflammation, endothelial dysfunction and micro- and macrovascular complications in patients of T2DM [35,36]. In a study by Agardh and Gomez based on animal model of diabetic retinopathy, upregulated VCAM-1 expression in blood vessels was observed in the retina under hyperglycemic conditions [37]. A similar observation was made in a study by Hegazy et al., where serum levels of soluble VCAM were found to be significantly upregulated in diabetic patients vs. healthy controls [38]. A larger cohort study reported that plasma levels of VCAM were significantly upregulated in patients of Diabetic Kidney disease [39]. Various studies have shown that the siRNA-mediated downregulation of Glo-1 perpetuated an increase in the expression of VCAM-1 in endothelial cells. Similarly, the overexpression of Glo-1 under diabetic conditions has also been reported to induce the expression of VCAM-1 [40].

TXNIP wears many different hats; it is an endogenous inhibitor of the thioredoxin (TRX) system, a major cellular thiol-reducing and antioxidant system; it regulates metabolism as well as inflammatory and angiogenic processes and also acts as a antiproliferative or proapoptotic agent [41]. It also interacts with the NLRP3 inflammasome to bring about an inflammatory storm via the production of IL-1β and eventually leads cells toward pyroptosis; this event is thought to be the ultimate link between inflammation and oxidative stress [42]. TXNIP levels are reported to be elevated in diabetic patients and under hyperglycemia, suggestive of the fact that TXNIP not only regulates the onset of diabetic vascular complications due to its involvement in hyperglycemia and oxidative stress, but also plays a role in their progression [18]. Our results suggest that Glo-1 and TXNIP might be partners-in-crime mediating hyperglycemia and the oxidative stress-driven onset and progression of micro- and macrovascular complications in T2DM. Interestingly, a study conducted on type-1 diabetic rats reported a concomitant upregulation of both VCAM and TXNIP accompanied by increased ROS levels [43]. This is an indication that Glo-1, VCAM and TXNIP are upregulated in hyperglycemia leading to oxidative stress, and mediate endothelial dysfunction, eventually causing vascular complications in T2DM. 

## 5. Conclusions

Our study demonstrates that the modulation of Glo-1 underlying dicarbonyl stress leading to hyperglycemia preceding vascular complications correlates with the expression of VCAM and TXNIP in micro- and macrovessels. VCAM is upregulated by both dicarbonyl stress as well as diabetic conditions. The expression of TXNIP is upregulated in the case of diabetic conditions, whereas TXNIP levels remain unperturbed under dicarbonyl stress. 

## Figures and Tables

**Table 1:** Primer sequences used for gene-expression profiling via real-time quantitative PCR.

Gene	Organism	Forward Primer (5′ to 3′)	Reverse Primer (5′ to 3′)
Glo-1	Human	GGTTTGAAGAACTGGGAGTCAAA	ATCCAGTAGCCATCAGGATCTTG
Nrf-2	Human	TCATGATGGACTTGGAGCTG	CATACTCTTTCCGTCGCTGA
IL-1β	Human	AATCTGTACCTGTCCTGCGTGTT	TGGGTAATTTTTGGGATCTACACTCT
SOD-2	Human	CAGGACCCACTGCAAGGAA	CGTGCTCCCACACATCAATC
SIRT-1	Human	CCGCGGATAGGTCCATATACT	AACAATCTGCCACAGCGTCA
VCAM-1	Human	ACATGGAATTCGAACCCAAA	TGTATCTCTGGGGGCAACAT
ICAM-1	Human	CCTTCCTCACCGTGTACTGG	AGCGTAGGGTAAGGTTCTTGC
NLRP-3	Human	GATCTTCGCTGCGATCAACA	GGGATTCGAAACACGTGCATTA
Caspase-1	Human	GCCTGTTCCTGTGATGTGGAG	TGCCCACAGACATTCATACAGTTTC
TXNIP	Human	TGGTGGATGTCAATACCCCT	ATTGGCAAGGTAAGTGTGGC
β_2_-Microglobulin	Human	GGCTATCCAGCGTACTCCAAA	CGGCAGGCATACTCATCTTTTT
Cyclophilin	Human	TTCCTGCTTTCACAGAATTATTCC	GCCACCAGTGCCATTATGG
Glo-1	Rat	GAAGATGACGAGACGCAGAGTTAC	CAGGATCTTGAACGAACGCCAGAC
Nrf-2	Rat	ACTCCCAGGTTGCCCACAT	CATGGTCATCTACAAATGGGAATG
IL-1β	Rat	CTGCAGCTGGAGAGTGTGGAT	CACTTTGCTCTTGACTTCTATCTTGTTG
SOD-2	Rat	CAGGACCCACTGCAAGGAA	CGTGCTCCCACACATCAATC
SIRT-1	Rat	CCGCGGATAGGTCCATATACT	AACAATCTGCCACAGCGTCA
VCAM-1	Rat	AGTGTGAATCGAAAACCGAAGTC	AATGGCGGGTATTACCAAGGA
ICAM-1	Rat	GCTGCGCTGTGTTTTGGA	GGATGGGAGCTGAAAAGTTGTAGA
NLRP-3	Rat	AAGCTGTCCTCAGTCAGAGGCAAT	ATGACTTTCTTGGAGCCAGGGACA
Caspase-1	Rat	AGGAGGGAATATGTGGG	AACCTTGGGCTTGTCTT
TXNIP	Rat	CAGACCTCCAAGACCACGACTG	CATCCGCAGCCAATGAACAGAG
β_2_-Microglobulin	Rat	TGGCCGTCGTGCTTGCCATT	TCTCCGGTGGATGGCGAGAGT
Cyclophilin	Rat	TTCCTCCTTTCACAGAATTATTCCA	CCATTATGGCGTGTGAAGTCA

## Data Availability

All data generated during this study are included in the article.
